# Affective empathy predicts self-isolation behaviour acceptance during coronavirus risk exposure

**DOI:** 10.1038/s41598-021-89504-w

**Published:** 2021-05-12

**Authors:** Serena Petrocchi, Sheila Bernardi, Roberto Malacrida, Rafael Traber, Luca Gabutti, Nicola Grignoli

**Affiliations:** 1 Institute of Communication and Health, Università della Svizzera Italiana, Lugano, Switzerland; 2 Laboratory of Applied Psychology and Intervention, Department of History Society and Human Studies, Università del Salento, Lecce, Italy; 3 Sasso Corbaro Medical Humanities Foundation, Bellinzona, Switzerland; 4 Cantonal Sociopsychiatric Organisation, Public Health Division, Department of Health and Social Care, Repubblica e Cantone Ticino, Mendrisio, Switzerland; 5 Department of Internal Medicine, Regional Hospital of Bellinzona and Valleys, Ente Ospedaliero Cantonale, Bellinzona and Università della Svizzera Italiana, Lugano, Switzerland

**Keywords:** Psychology, Human behaviour, Infectious diseases, Psychiatric disorders, Health policy, Public health

## Abstract

Health risk exposure during the global COVID-19 pandemic has required people to adopt self-isolation. Public authorities have therefore had the difficult task of sustaining such protective but stressful behaviour. Evidence shows that besides egoistic drives, the motivation for self-isolation behaviour could be altruistic. However, the type and role of prosocial motivation in the current pandemic is underestimated and its interaction with risk exposure and psychological distress is largely unknown. Here we show that affective empathy for the most vulnerable predicts acceptance of lockdown measures. In two retrospective studies, one with a general population and one with COVID-19 positive patients, we found that (1) along with health risk exposure, affective empathy is a predictor of acceptance of lockdown measures (2) social covariates and psychological distress have no significant impact. Our results support the need to focus on altruistic behaviours while informing the public instead of on fear-inducing messages.

## Introduction

Lockdowns (namely collective physical distancing or isolation during a health emergency) have been necessary, besides mass-vaccination, for flattening the curve of the COVID-19 infection worldwide^1^ but have a detrimental impact on global mental health^2^. During the current pandemic psychological science has gained the spotlight^3^, offering valuable understanding on how public authorities can mitigate such a stressful condition and at the same time help individuals to manage risk while maintaining motivation for self-protective and prosocial behaviours^4^.

The impact of public health measures on individual well-being and psychosocial functioning was one of the factors investigated in the course of behavioural management during previous pandemics. Review of these studies revealed a negative psychological impact generated by physical isolation and quarantine^5,6^. This evidence from measures taken for mixed lists of diseases that are prioritized in the context of public health emergencies tends to be confirmed by research on confinement conducted during the COVID-19 outbreak^7,8^. It has been acknowledged that isolation in the current public health crisis is a major stress factor that will contribute to an increased risk of psychiatric illness^9,10^. Stress-related disorders such as PTSD, anxiety or insomnia were observed in the early phases of COVID-19 and could be considered an expression of specific health anxiety in reaction to the risk of contagion during the pandemic outbreak^11^. Such a COVID-19 fear reaction has been described as coronaphobia, an emotional state that might induce exaggerated help-seeking behaviours during the pandemic^12^. Depressive symptoms have also been observed^13^ and their increase is expected if the physical distancing measures are maintained in the long term^14^. Maintaining physical distance over a long period would in fact increase loneliness, which is recognized as a risk factor for mental health and in particular for depressive disorders of the elderly and middle aged^15^.

This emerging evidence from literature has enriched the debate on what aspects may protect individuals from the negative side effects of lockdown during a pandemic and at the same time promote compliance with public health rules. People’s willingness to comply with preventive public health behaviours is in fact known to be associated with an interaction between risk exposure/perception^16^ and various psychosocial factors^3,17^. Among the psychological factors modulating both risk perception and preventive health behaviours, prosocial values may play a central role^18^ and empathy appears to be in the forefront. In fact engaging in those behaviours both protects oneself and at the same time the most vulnerable, who may be approached through the different constitutive components of empathy, in particular affective response (or sympathetic/empathic concern), the perspective-taking cognitive process and regulatory mechanisms necessary to distinguish self- from other-feelings^19^. Empathy, defined as a process of sharing another person’s emotion and understanding their emotional state, has been described in a recent narrative review as effective in intergroup conflict resolution and valuable for targeted group intervention^20^. A study conducted in the US during the COVID-19 epidemic peak^21^ showed that compared to messages that induce fear, prosocial messages capable of arousing a positive emotional state have proved to be more effective in the willingness to accept self-isolation. Recent studies on the general population suggested that prosocial mental attitudes, such as affective empathy, might promote compliance to public health rules^22–24^.

Other research conducted during the COVID-19 outbreak indicates, however, that empathy levels might fluctuate according to anxiety linked to risk exposure/perception and modulate prosocial willingness^25,26^. Perceived risk may be relative to the individual (i.e. one’s health) or to third parties (i.e. being a danger to others), which can be experienced by both infected and non-infected individuals. Altruistic acceptance of risk has been previously identified as a factor reducing the psychological burden on healthcare professionals during a pandemic, in particular in diminishing post-traumatic and depressive symptoms^27,28^. Notwithstanding, empathic abilities are recognized from a developmental point of view as associated with emotional distress and could be seen as “risky strengths”^29^.

To our knowledge, there are no studies to date that have analysed whether people’s affective empathy, interacting with the level of risk exposure and health anxiety, influences compliance with public health rules during the current COVID-19 pandemic. Furthermore, none of the screened literature has investigated those psychological factors in people affected or hospitalized for COVID-19: such data are hard to obtain but greatly needed for better informing public health decision-makers. Therefore, the main aim of the present research was to explore whether several psychological factors (i.e. affective empathy, risk exposure condition, psychological distress) increase acceptance of the COVID-19 pandemic lockdown. To this end, the study was developed in two distinct phases. Through a retrospective design involving the general population under the mandatory lockdown for COVID-19, Phase 1 explored whether a high level of affective empathy is a determinant of the acceptance of the lockdown, controlling for psychological distress, health status and socio-demo characteristics (i.e. RQ1). Phase 2 added the evaluation of the risk exposure condition in terms of being part of one of three groups: COVID positive/hospitalised individuals (high risk) *vs* COVID positive/home isolated individuals (moderate risk) *vs* COVID negative/home isolated individuals (low risk). Specifically, Phase 2 tested whether affective empathy is a significant predictor of acceptance of the lockdown, even controlling for psychological distress and considering the three risk conditions (RQ2).

## Results

Regarding data collected in Phase 1, descriptive statistics for the socio-demo are reported in Table 1.

**Table 1:** Demographics of Phase 1 and Phase 2.

	Phase 1	Phase 2			
	COVID negative/home isolated individuals (N = 339)	COVID positive/hospitalized individuals (A) (N = 76)	COVID positive/home isolated individuals (B) (N = 63)	COVID negative/home isolated individuals (C) (N = 61)	Comparisons
**Age** M and (SD)—(range)	41.81 (13.48)—(18–80)	62.13 (14.76) – (24–87)	41.90 (14.25) – (19–75)	41.31 (13.57) – 19–74)	F^a^(2, 197) = 48.90***
**Sex** female:male	289:50	20:56	38:25	51:10	χ^2b^ (2) = 46.06***
**Marital Status %**					
Married	43.9	69.7	41.3	40.9	χ^2c^ (2) = 27.25***
Single	39.5	13.2	42.9	44.3	
Divorced/Separated/Widowed	14.8	17.1	15.9	14.8	
**Occupation %**					
Employed	71.3	44.7	85.7	72.1	χ^2d^ (2) = 15.45***
Homemaker	1	–	–	–	
Unemployed	8.6	5.3	1.6	4.9	
Student	11.7	1.3	6.3	18	
Retired	7.4	48.7	6.3	4.9	

SD = standard deviation; M = mean; * = p < .05; ** = p < .01; *** = p < .001.

^a^ = A group had age means higher than the other two groups that were not different from each other.

^b^ = A group had more men than the expected, while C group had more women than the expected.

^c^ = Comparisons based on risk groups and marital status dummy coded (presence vs. absence of a partner): high-risk group had a partner more than the expected.

^d^ =  Comparisons based on risk groups and occupation dummy coded (employed vs. unemployed): high-risk group had no occupation more than the expected.

Descriptive showed that, respectively, 59.6% and 50.7% of the participants reported low anxiety and depression symptoms under the cut-off score of 5. On the other hand, 40.4% of the participants reported from mild to severe anxiety symptoms and 49.3% from mild to severe depression symptoms.

Non-parametric comparisons between females and males of their affective empathy, depression, anxiety and acceptance of the lockdown were conducted. No differences emerged from these analyses. Results of the correlations revealed that younger participants showed less anxiety, depression and distress. Sex showed no relationships with the other variables, whereas the household composition was negatively associated with the acceptance of the lockdown. Participants who had a partner showed less depression, while those who had an occupation displayed less depression and anxiety. The question on general health was negatively correlated with the number of chronic diseases, affective empathy, depression, anxiety, distress and acceptance of the lockdown. Contrarily, the correlations between those variables and the number of chronic diseases showed a positive sign: affective empathy, anxiety, depression and distress were positively correlated with each other. Acceptance of the lockdown correlated negatively with general health, and positively with the number of chronic diseases and affective empathy. Table 2 shows details of the results.

**Table 2:** Correlations, Phase 1.

	M, range	SD	Sex	Household composition	Partner	Occupation	General health	Chronic diseases	Affective Empathy	Depression	Anxiety	Distress	Acceptance of lockdown
Age	41.81 18–80	13.48	.001	−.15**	.35***	.003	.14*	.05	.01	−.25***	−.21***	−.19**	.05
Sex^1^ (0 = males) (1) females:males	289:50	–		.06	.06	.01	.003	.04	.07	.01	.06	.10	−.005
Household composition (2)	2.69 0–6	1.2			.42***	.04	.004	−.19***	−.11*	.05	.08	.03	−.13*
Partner^1^ (0 = no) (3) yes:no	172:228	–				.16*	.06	−.09	−.02	−.15*	−.08	−.06	−.01
Occupation^1^ (0 = no) (4) yes:no	283:117	–					.12*	−.23***	−.07	−.17**	−.12*	−.09	−.10
General health (5)	3.79 1–5	.91						−.42***	−.17**	−.31***	−.27***	−.21***	−.13*
Chronic diseases (6)	.089 0–6	1.15							.15**	.21***	.19***	.15**	.12*
Affective empathy (7)	4 1–5	.93								.12*	.20***	.25***	.54***
Depression (8)	8.83 1–25	5.49									.85***	.68***	.05
Anxiety (9)	5.47 0–21	4.85										.70***	.06
Distress (10)	5.7 1–10	2.7											.08
Acceptance of lockdown (11)	3.85 1–5	.95											

*p < .05. **p < .01, *** *p* < .001;

^1^ = point biserial correlations were calculated.

**Figure 1:**
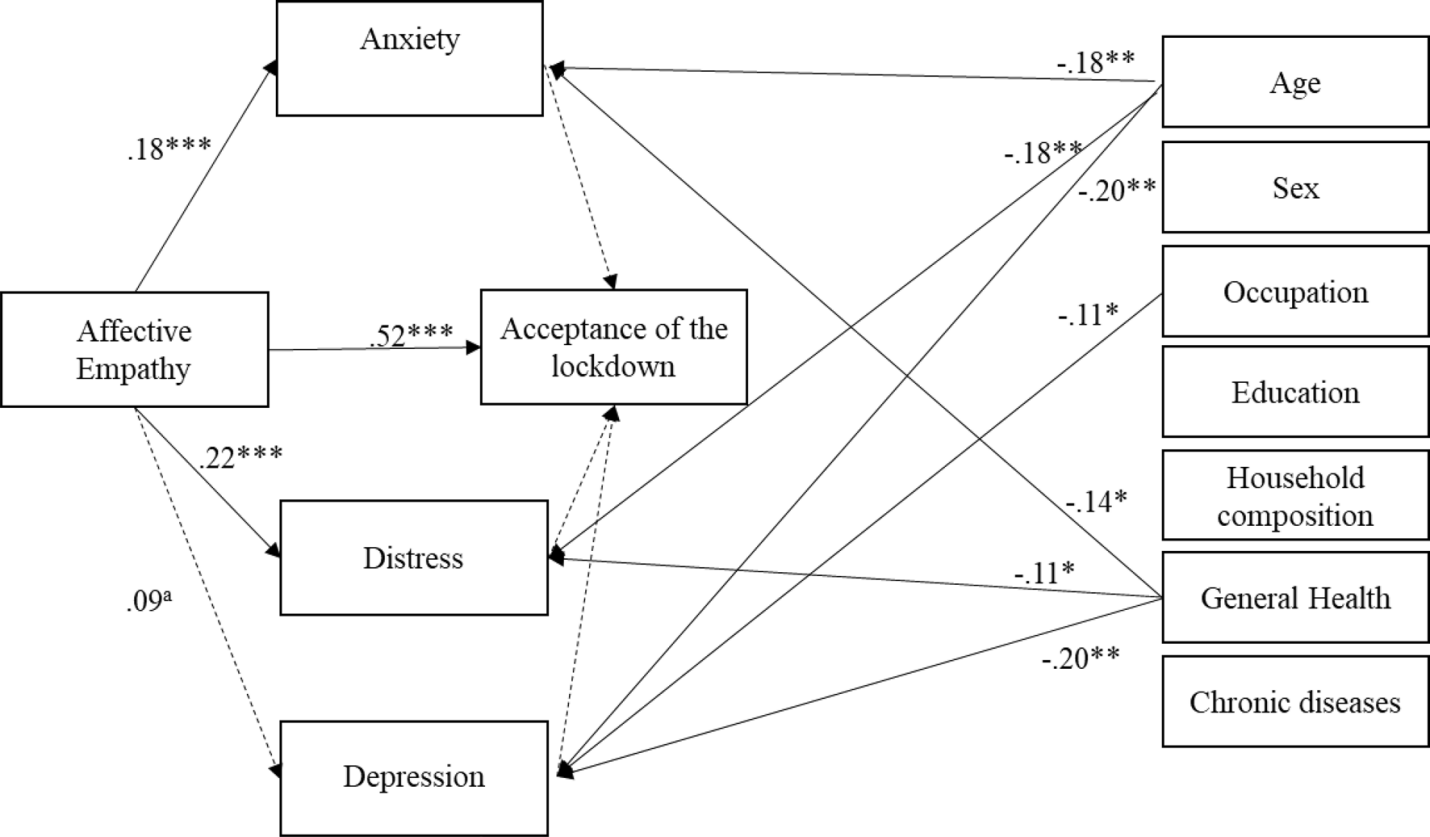
Mediation Analysis, Phase 1. Note: for readability purpose, only significant paths between covariates and the other variables have been drawn. ^a^ = .08, * = p < .05; ** = p < .01; *** = p < .001.

Mediation analysis yields various similar results. First, an overall path led from affective empathy to acceptance of the lockdown. Secondly, depression, anxiety and distress were not significant mediators in the relationship between affective empathy and acceptance of the lockdown. In other words, individuals with high levels of affective empathy were inclined to accept the lockdown over and above the perception of psychological distress. Affective empathy was significantly related with anxiety and distress, but not with depression. Thirdly, the covariates (i.e. age, sex, household composition, general health, chronic diseases, occupation and education) were not significantly associated with acceptance of the lockdown. Younger participants showed greater anxiety, distress and depression. The unemployed showed greater depression, while lower levels of perceived general health were linked to higher anxiety, distress and depression. Figure 1 shows the graphical representation of the mediation analysis. The indirect effects were not significant, whereas the total effect (β = 0.51, SE = 0.05, LLCI = 0.41, ULCI = 0.62) and the direct effect (β = 0.53, SE = 0.05, LLCI = 0.42, ULCI = 0.63) were significant. These results confirmed that between psychological distress and affective empathy, the latter led to a greater acceptance of isolation as a behavioural norm to fight the threat of COVID-19.

**Figure 2:**
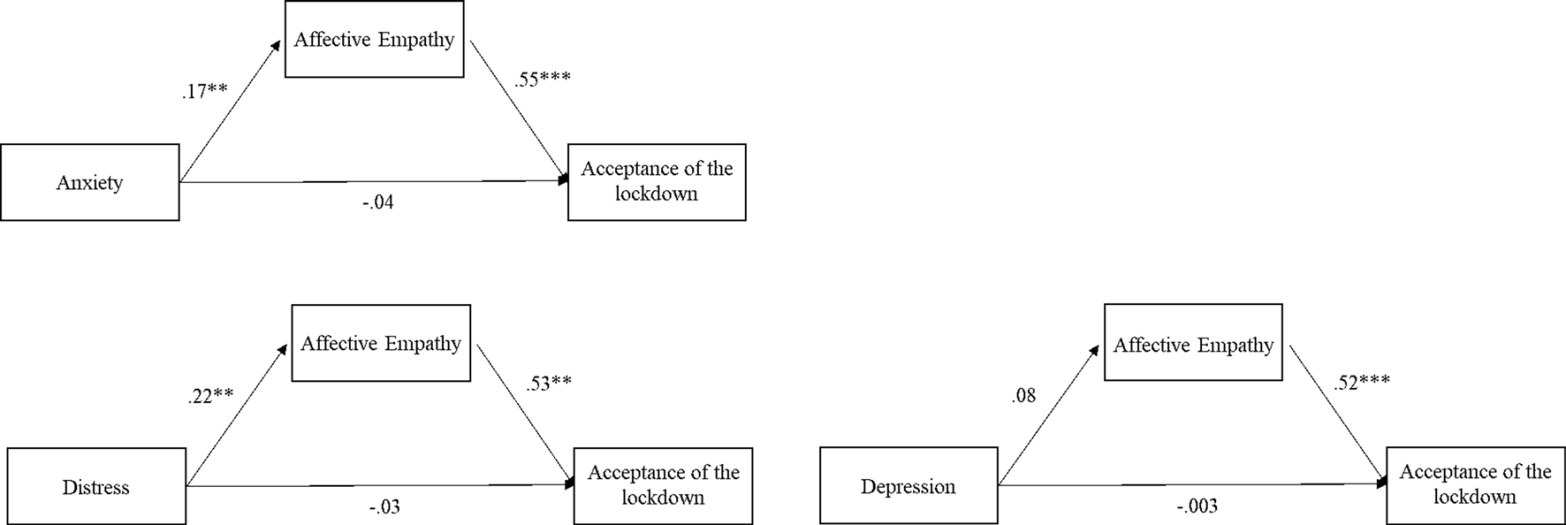
Mediation analyses, reverse models, Phase 1. Note: The covariates are not listed for readability purpose. * = p < .05; ** = p < .01; *** = p < .001.

Since the research had a retrospective design, reverse models were tested. Model 1 tested whether anxiety was significantly directly associated with acceptance of the lockdown and through the mediation of affective empathy. Models 2 and 3 tested similar relationships of affective empathy and acceptance of the lockdown with distress and depression, respectively. Figure 2 shows the results of the three models. Affective empathy was a significant mediator in the relationships between psychological distress variables and the acceptance of the lockdown, whereas no direct effects were found between psychological variables and the outcome. The indirect effects were significant for Model 1 β = 0.10, SE = 0.03, LLCI = 0.02, ULCI = 0.16, and Model 2 β = 0.17, SE = 0.03, LLCI = 0.05, ULCI = 0.19, but not for Model 3. The total effects and direct effects were not significant. The reverse model describes a bidirectional relationship between affective empathy and distress in determining the acceptance of the lockdown.

Regarding data collected in Phase 2, descriptive statistics for the socio-demo are reported in Table 1. Preliminary ANOVAS showed significant differences on distress, depression and anxiety when the three groups of participants were compared (i.e. COVID positive/hospitalised individuals; COVID positive/home isolated individuals; COVID negative/home isolated individuals). The post hoc comparisons using the Duncan test indicated that the mean scores of distress and anxiety for the COVID positive/hospitalised individuals were significantly higher than the scores of the COVID negative/home isolated individuals. However, the scores of the COVID positive/hospitalised individuals and COVID positive/home isolated individuals did not differ significantly. As for depression, the scores of the three groups were all significantly different from each other, with the COVID positive/hospitalised individuals showing higher scores. Table 3 gives details of the results. Similarly, the ANOVAS comparing the three groups of risk showed significant differences in affective empathy and acceptance of the lockdown. The post hoc test revealed that the mean score of both variables was significantly higher in the COVID positive/hospitalised individuals and COVID positive/home isolated individuals than in the COVID negative/home isolated individuals; the COVID positive/hospitalised individuals and the COVID positive/home isolated individuals did not show a significant difference. See Table 3 for details.

**Table 3:** Results of the ANOVA.

	**F(df)**	**M (SD)**		
		**COVID positive/hospitalized individuals (A)**	**COVID positive/home isolated individuals (B)**	**COVID negative/home isolated individuals (C)**
**Distress**	F(2 177) = 5.8**	6.73^a^ (2.4)	6.37 (2.2)	5.18 (2.8)
**Anxiety**	F(2 192) = 12.64***	7.5 ^a^ (5.3)	6.5 (4.7)	4.5 (4.8)
**Depression**	F(2 192) = 5.9**	11.2^b^ (6.6)	8.9 (5.8)	5.8 (5.7)
**Affective empathy**	F(2 194) = 7.2***	4.3^c^ (.8)	4.4 (.6)	3.8 (1)
**Acceptance of the lockdown**	F(2 194) = 13.68***	4.3^c^ (.68)	4.3 (.5)	3.6 (.9)

*p < .05. **p < .01, *** *p* < .001.

^a^ = Significant Post-Hoc comparisons: A group > C group;

^b^ = Significant Post-Hoc comparisons: A group > B group > C group;

^c^ = Significant Post-Hoc comparisons: A group and B group > C group.

Non-parametric comparisons between females and males on their affective empathy, depression, anxiety and acceptance of the lockdown were conducted. No differences emerged from these analyses. Results of the correlations revealed that younger participants showed less depression. sex showed no relationship with the other variables, whereas the household composition was negatively associated with the acceptance of the lockdown. The question on general health was negatively correlated with the number of chronic diseases, depression and anxiety. Contrarily, the correlations between depression, distress and number of chronic diseases showed a positive sign. Affective empathy, anxiety, depression and distress were positively correlated with each other. The acceptance of the lockdown positively correlated with age, affective empathy, depression, anxiety and distress, and negatively with household composition. Table 4 shows details of the results.

**Table 4:** Correlations, Phase 2.

	M, range	SD	Sex	Household composition	Partner	Occupation	General health	Chronic diseases	Affective Empathy	Depression	Anxiety	Distress	Acceptance of lockdown
Age	49.41 19–87	17.33	−38***	−34***	−.43***	−.35***	−14	.26***	.05	.16*	.08	.03	.16*
Sex^1^ (0 = males) (1) females:males	109:91	–		.16*	−.11	.26***	.10	−.19**	−.02	−.08	−.02	−.02	−.09
Household composition (2)	2.69 0–6	1.2			.25***	.06	.09	−.13	−.15*	−.06	.06	.02	−.17*
Partner^1^ (0 = no) (3) yes:no	132:68	–				−.04	−.02	.007	.04	.05	.09	.001	.06
Occupation^1^ (0 = no) (4) yes:no	104:96	–					.26***	−.18***	−.08	−.25***	−.14	−.17*	−.06
General health (5)	3.79 1–5	1						−.53***	−.03	−.34***	−.24**	−.14	−.02
Chronic diseases (6)	1.11 0–11	1.55							−.05	.20**	.10	.16*	.06
Affective empathy (7)	4.19 1–5	.86								.27***	.27***	.37***	.64***
Depression (8)	8.81 1–26	6.48									.82***	.64***	.29***
Anxiety (9)	6.23 0–21	5.16										.63***	.26***
Distress (10)	6.18 1–10	2.56											.26***
Acceptance of lockdown (11)	4.09 1–5	.90											

*p < .05. **p < .01, *** *p* < .001.

^1^ = point biserial correlations were calculated.

**Figure 3:**
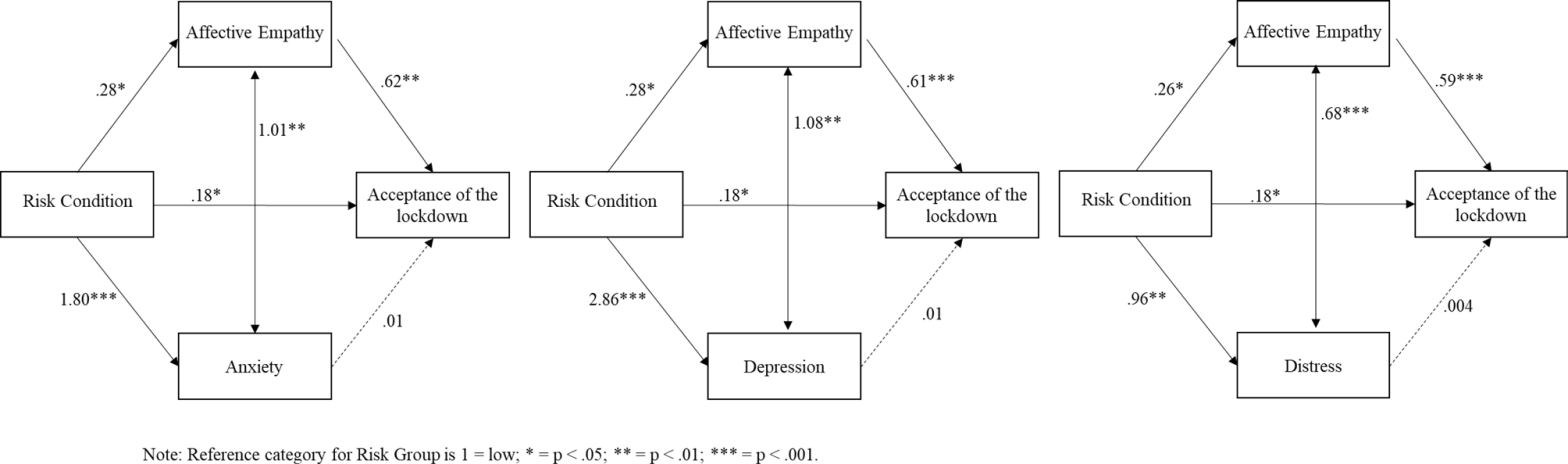
The models tested, Phase 2.

The three tested models showed a good fit with the data, χ^2^ (2) = 107.57, p < 0.001, CFI = 1, RMSEA = 1, for the model with anxiety, χ^2^ (2) = 140.89, p < 0.001 CFI = 1, RMSEA = 1 for the model with depression, and χ^2^ (2) = 98.50, p < 0.001, CFI = 1, RMSEA = 1 for the model with distress. In all three models, the higher the objective risk the greater was the anxiety, depression and distress experienced by participants and the higher was the affective empathy. Affective empathy was a mediator in the relationship between objective risk and acceptance of the lockdown. Therefore, individuals with high levels of affective empathy perceived the lockdown as necessary for overcoming COVID-19. Finally, there was a direct effect of risk on the acceptance of the lockdown: the worse the risk, the higher the perception of acceptance of the lockdown. The three indirect paths from objective risk through affective empathy confirmed the above results, *β* = 0.17, *p* = 0.016 for anxiety, *β* = 0.17, *p* = 0.016 for depression, and *β* = 0.15, *p* = 0.03 for distress. The corresponding total effects were significant as well, *β* = 0.36, *p* < 0.001 for anxiety, *β* = 0.35, *p* < 0.001 for depression, and *β* = 0.34, *p* = 0.001 for distress. The indirect paths from objective risk through the psychological variables were not significant, *β* = 0.02, *p* = 0.25 for anxiety, *β* = 0.03, *p* = 0.15 for depression, and *β* = 0.004, *p* = 0.84 for distress. The total effects, considering anxiety, depression and distress, were significant (*β* = 0.21, *p* = 0.01 for anxiety, *β* = 0.21, *p* = 0.01 for depression, and *β* = 0.19, *p* = 0.02 for distress). See Fig. 3 for details of the models.

Given the cross-sectional design of our study, the risk exposure condition could be a moderator variable in the relationship between empathy and acceptance of the lockdown. We tested this relationship and found a significant model, F(5 191) = 33.93, p < 0.001, R2 = 0.47, however the test of the highest order unconditional interactions was not significant, F(2 191) = 2.85, p = 0.06, Rchange2 = 0.01, meaning that the interaction terms between empathy and risk conditions did not add any significant part of the variance in the explanation of the outcome and that the subsequent results should not be considered.

## Discussion

The emerging evidence from literature has stressed that lockdowns can exert a detrimental effect on an individual’s mental health. Thus, the question for public health professionals and experts is: what aspects may protect individuals from the negative side effects of lockdown during a pandemic and, at the same time, promote compliance with public health measures? This question leads to the need to understand how individuals are motivated to protect themselves and others^30,31^. The results of the present research contribute to the debate by providing answers related to the role of affective empathy, objective risk exposure and the psychological impact of lockdown during a pandemic.

Phase 1 found that the more an individual experienced affective empathy regarding the most vulnerable people, the more they perceived the lockdown as an effective measure and the more they experienced anxiety, depression or distress. However, the psychological impact of COVID-19 was not associated with the acceptance of the lockdown. Even the presence of chronic diseases, which could be considered a proxy for objective health risk condition, was not associated with the acceptance of the lockdown. In other words, individuals with high levels of affective empathy were inclined to accept the lockdown over and above their perception of psychological distress and their risk condition. This is consistent with previous studies showing that affective empathy is a prosocial attitude increasing adherence to physical distancing and hygiene measures during the pandemic^22–24^. Moreover, our results show that the role of a prosocial disposition, like affective empathy, is more effective than other negative psychological outcomes such as anxiety/worry and emotional distress. These are recognized in the literature as related protective behaviours in the pandemic^32^.

Phase 2 added the evaluation of the risk exposure condition in the forms of being included in one of the three groups: COVID positive/hospitalized individuals *vs.* COVID positive/home isolated individuals *vs.* COVID negative/home isolated individuals. Therefore, Phase 2 considered a more accurate measure for testing the risk exposure condition of the three different groups of participants according to their COVID-19 exposure and symptomatology. The aim was to test whether affective empathy remained a significant predictor of the acceptance of the lockdown, considering the specific risk exposure condition related to COVID-19 infection and psychological distress. The results demonstrated that, as expected, being COVID positive/hospitalised individuals was associated with greater anxiety, depression and distress. These results are consistent with international literature^9,11^ and those from the same geographical region as our study^33^. Moreover, even though the risk exposure condition was significantly associated with the acceptance of the lockdown (i.e. the higher the risk the greater the acceptance), affective empathy was still a significant predictor, even controlling for psychological distress. We expected that the risk exposure condition would play a role in determining both psychological distress and the acceptance of lockdown. The originality of this research is given by confirmation that individuals who expressed concern for the vulnerable (i.e. affective empathy) were more willing to accept the lockdown measures that are known to create distress, even taking into account their risk exposure.

According to the evidence of our research, we would propose some practical implications regarding how to direct public health communication. One possibility is that communication at a time of health crisis may induce people to protect themselves and others through fear. Simple public health messages focusing on health risk may induce fear of contagion but may also have detrimental consequences for psychological distress and collective well-being, without increasing adherence to self-protective behaviours necessary to contain the virus spread. Evidence from the use of fear as a means in communication is inconsistent and often highlights a boomerang effect^34^.

A current debate acknowledges the importance of reducing the psychological impact related to uncertainty and fear of infection experienced during a pandemic. More complex messages targeting a peer group sharing risk awareness that focuses on the protection of the vulnerable could be envisaged. In doing so, communicators should express their empathy, demonstrating concern and understanding regarding the impact of the pandemic on individual lives^35^. The results of the present research are to be placed within this debate, with the potential to add several practical considerations.

Evidence collected during the present pandemic shows that messages focussing on the collective threat (i.e. family or loved ones, community) promote the intention to comply with public health rules (e.g. face covering^36^ or eliciting clickthroughs on official information^37^). Moreover, compared to messages that induce fear, prosocial messages capable of arousing a positive emotional state are more effective in inducing willingness to accept self-isolation^21^. In line with evidence collected in the same country as this study^33,38^, our results demonstrate that when the risk for an individual’s health and survival is high, the level of psychological negative impact is high as well. However, a negative psychological impact did not lead to a greater acceptance of the quarantine. In this sense, individuals might be more inclined to respect the lockdown if the communication stresses the risk for vulnerable people being infected by a virus that spreads at an exponential rate^39^; this provokes their empathic tendencies (i.e. affective empathy). Our results actually demonstrate that affective empathy, along with risk exposure condition, is one of the possible mechanisms that reinforce the acceptance of measures that restrict individual freedom. Risk exposure condition, however, cannot be manipulated for communication purposes, whereas affective empathy can. Recent studies have shown that brief interventions aimed at the mechanism of psychological flexibility (and indirectly at values such as caring and supporting others) lead to increased prosocial behaviours in the laboratory and in everyday life^40^.

Therefore, as public health communication has more influence on people when individuals perceive that the communicator is empathetic^35^, similarly we propose to use a form of peer communication and to target the message by including calls for affective empathy. Such a call is particularly needed when considering a potential effect of a decline in empathy due to the persistence of the pandemic^26^. The target for this kind of intervention should be people with low risk because they are less likely to be inclined to adopt self-isolation measures. Sex and gender issues may influence COVID-19 effective risk exposure^41^ and negative psychological impact or psychological and behavioural reaction during the pandemic^13,42^, and should be taken into account for targeted interventions. In particular, evidence shows that women score higher than men in trait empathy (measured by the Interpersonal Reactivity Index) during the pandemic and suffer more than men from depression, anxiety and trauma^43^. Furthermore, gender has been proved an influential factor in compliance with public health rules during the COVID-19 pandemic. For example, an experiment testing the effect of messaging on prevention measures showed that men are less prone to wearing a face covering and feel less at risk than women of being seriously affected by the coronavirus^36^. Such targeted risk groups, after training, could become official communicators. In this way they would strengthen a social connection that has been shown to be a major factor in mitigating the negative psychological impact of the lockdown^44^. Our proposal is in line with recent claims in this direction that highlight shared prosocial motivation as one of the global lessons to be learned from the COVID-19 pandemic^45^.

Our study opens different future perspectives in the field of prosocial human behaviour during pandemics. Replication of this study with a validated psychometric measure of affective empathy and acceptance of public health rules could improve the understanding of the psychological processes involved in the impact of such prosocial attitudes on mental health. Previous work on prosocial attitudes in healthcare professionals shows for example that perspective-taking (i.e. cognitive empathy) is preeminently protective of burnout risk if that cognitive process is not overwhelmed by emotional contagion (anxiety) in a threatening situation^46,47^. The protective role of cognitive processes of empathy on prosocial behaviour in interpersonal relations is in fact recognized in the current literature^20^ and evidence gathered during the current pandemic tends to confirm this tendency. An online study of students and the general population found a positive correlation between trait empathy (measured by the Interpersonal Reactivity Index), particularly perspective-taking subscale, and social distancing tendencies^24^. Another recent online study on the general population conducted in Germany tends to confirm our data and shows how affective empathy, empathy for “loved ones” and moral norms are related to self-elicited social distancing behaviour^23^. The perspective-taking process at stake in empathy could also be linked to the subsequent process of distinction between self and the other (namely the “community” or “loved ones”). Identifying which cognitive or affective process is involved with an increase of prosocial behaviour, and how it will modulate psychological distress during risk exposure can be a major challenge for future studies aimed at informing public health communication during the current or future pandemics.

Data show that decreased psychosocial well-being is related to greater difficulty in adhering to physical distancing^48^. However, our results did not confirm these findings. In particular they did not show a reduced psychological impact for individuals with a higher acceptance of lockdown measures. This aspect needs to be investigated in further research with the aim of testing relationships among variables over time and establishing proper causal effects between them.

During a highly infective pandemic, we have learned the most important protective behaviours – respect the lockdown, wash your hands, cough in a tissue, keep distance, wear a mask, stay isolated if COVID-19 positive – but we have also realized that how communication fosters those behaviours is not yet fully defined. Our evidence discourages insisting on fear while informing the population, but rather suggests that inducing concern for the vulnerable (i.e. affective empathy) may be a way to promote adherence to behavioural measures of prevention and to target specific interventions to reduce the psychological burden.

## Methods

### Procedure

In both Phase 1 and Phase 2, participants were asked to refer retrospectively to the period from 22 March to 11 May 2020 during the mandatory lockdown in Switzerland. From the end of February to the end of June 2020, the Swiss Federal Government implemented mandatory measures of quarantine and isolation. In Phase 1, participants from the general population replied to an online survey via QualtricsTM. They were recruited from 8 September to 15 October 2020. The research was repeatedly advertised on the Facebook page of the University. Participants did not receive any compensation and the questionnaire was completed in approximately 15–20 minutes.

In Phase 2, the data collection took place between 4 August and 5 October 2020. Patients were recruited through the local hospital database on COVID-19 cases provided by Ente Ospedaliero Cantonale (EOC). Individuals were recruited by phone and could decide between the paper–pencil questionnaire or the online version.

This research project was conducted in accordance with the protocol^49^, the Declaration of Helsinki, the principles of Good Clinical Practice, the Human Research Act (HRA) and the Human Research Ordinance (HRO), as well as other locally relevant regulations. The study was approved by the Cantonal Ethical Committee (N. 2020–01,460 /CE3679). Participation was voluntary and data collection was in an anonymous form. Participants received an information sheet and gave their informed consent for participation. Since the fact of evaluating their basic psychological status associated with lockdown might increase participants’ awareness of their mental health, a local public psychiatric organization number was included in the information sheet.

### Measures

#### Phase 1 and Phase 2

*Demographic.* Self-reported information on sex (i.e. male/female), age, marital status (i.e. presence/absence of a partner), occupation (i.e. employed vs unemployed), household composition (i.e. number of individuals in the household), was collected through specific questions.

*Previous health issues.* Self-reported previous diagnosis of non-COVID-19 diseases was collected through specific questions, as a proxy for objective health risk condition. The following diseases were detected: high blood pressure, heart problems; migraines, epilepsy, neurological diseases, stroke; vision or hearing problems; mental disorders or illnesses; diabetes, thyroid or other gland disorders; chronic infections; tumours; kidney diseases; diseases of the lungs; digestive diseases; addictions (alcohol, drugs, medicines). One point was attributed each time the participants claimed to suffer from a chronic disease. The final score was calculated as a sum of the participants’ answers with higher scores indicating worse health conditions.

*General Health*. An item evaluating general health as reported by participants was administered (“How would you rate your overall health compared to others your age?”). Response options were on a 5-point Likert scale from 1 (“very bad”) to 5 (“very good”).

*Affective Empathy*. This was measured by means of three items developed and tested in six related studies^50^ and then applied in a research on COVID-19^22^. These items were developed based on the empathic concern scale by Davis^51^. The items were back-translated and the specification “during the lockdown” was added at the beginning of each sentence. Therefore, the items applied in the present study were: “During the lockdown, I was very concerned about those most vulnerable to COVID-19”, “During the lockdown, I felt compassion for those most vulnerable to COVID-19”; “During the lockdown, I was quite moved by what could happen to those most vulnerable to COVID-19 infection”. Response options ranged from 1 (“strongly disagree”) to 5 (“strongly agree”). The items measuring empathic concern were mixed with three-filler items to reduce demand characteristics. The three items on empathic concern were averaged to create a final score (α = .84, rs > .63 [it was α > .81 in the original study^50^]) with higher scores indicating greater affective empathy.

*Acceptance of lockdown*. Four items evaluating participants’ acceptance of physical isolation (lockdown) were administered^52^. The items were: “The lockdown was very effective in stopping the spread of COVID-19”; “So many people were affected by COVID-19 that lockdown reduced the risk of infection”; “COVID-19 is highly contagious, so lockdown was useful”; “I don't understand why people have ignored lockdown restrictions”. Labels ranged from 1 (“strongly disagree”) to 5 (“strongly agree”). The final score was created as a mean of all the items (α = .85, rs > .45) with higher scores indicating greater acceptance of the lockdown.

*Psychological impact of lockdown.* The psychological impact of lockdown was investigated with the Italian version of the NCCN 11-point Likert scale Distress Thermometer, without the Problem List^53^. Anxiety symptoms were investigated through the Generalized Anxiety Disorder 7-item Scale (GAD-7)^54^ and depressive symptoms with the Patient Health Questionnaire-9 (PHQ-9)^55^. For GAD-7 and PHQ-9, participants indicated how often they had been troubled during lockdown by each symptom, using a four-point Likert scale ranging from 0 (“Not at all”) to 3 (“Nearly every day”). Two summative scores were created, with higher scores indicating greater anxiety or depression.

### Participants

#### Phase 1

According to Fritz and MacKinnon^56^, the sample size was estimated a-priori with a given power of 0.80. We estimated that both the effect from affective empathy to psychological distress, and from psychological distress to acceptance of the lockdown would be halfway between small and medium. The combination of the two effects led to a sample of 162 participants, since we applied a percentile bootstrap method for calculation. A total of 418 participants started the survey, 79 of whom failed to finish. No differences emerged between those who completed the survey only in part and those who finished, with the exception of affective empathy (U = 5280, p = 0.039). Participants who completed all the survey showed higher affective empathy (M = 3.87, SD = 0.95) compared to those who completed only part (M = 3.54, SD = 0.90). Table 1 presents the description of the final sample.

#### Phase 2

The sample size was estimated similarly to Phase 1. The effect from risk exposure condition to affective empathy and from affective empathy to acceptance of the lockdown were set as halfway between small and medium. This combination led to a sample of 162 participants. The effect from risk condition to psychological distress was set as large, whereas the effect from psychological impact to acceptance of the lockdown was set as halfway between small and medium. The combination of these two effects led to a sample of 123 participants.

The database provided by EOC included 500 patients over 18 years of age who had tested positive for COVID-19 and been hospitalized in isolation in post-acute phase between 22 March and 11 May 2020 (group 1). There were also 310 patients over 18 years of age who had tested positive for COVID-19 and been isolated at home between 22 March and 11 May 2020 (group 2). A third group of participants included individuals from the general population who were in lockdown during the same period and negative to COVID-19 infection. 195 randomly selected participants of group 1 were contacted by phone, 100 responded to the call and 90 gave their verbal consent to participate in the study (response rate 90%). For group 2, 192 randomly selected participants were contacted, 102 responded to the call and 90 gave their consent (response rate 88%).

Two-hundred participants aged from 19 to 87 (M = 49.41, SD = 17.33; 109 women) took part in the research. Participants were classified in three groups according to their risk exposure condition: a) group 1, COVID positive/hospitalised individuals (n = 76), group 2, COVID positive/home isolated individuals k (n = 63), group 3, COVID negative/home isolated individuals (low risk). (n = 61). Table 1 shows demographics of the final sample.

Non-parametric comparisons between phase I sample and phase II low risk sample were performed to assure that the two groups of participants were comparable. The comparisons showed a significance in their levels of depression, U = 9.937, p = 0.042, with participants in phase I showing higher levels of depression (M = 6.8, SD = 5.4) than participants in phase II (M = 5.8, SD = 5.7).

### Data analysis

#### Phase 1 and Phase 2

Analyses were carried out with SPSS v.26 and Rstudio. The normality distribution of the main variables was checked. Missing data did not exceed 7% in phase 1 and 10% in phase 2. The structural validity of the scale was tested through Reliability Analysis (Cronbach’s alpha and inter-item correlations). The mediation analysis in Phase 1 was performed through PROCESS macro v3.5 for SPSS v.26. Non-parametric tests were performed to test whether differences emerged between participants who completed the survey only partially or entirely. The Structural Equation Model in Phase 2 was tested using LAVAAN package or RStudio. The following goodness-of-fit indices were used to evaluate model-data correspondence: the Chi-square value, the Comparative Fit Index (CFI), the Root Mean Square Error of Approximation (RMSEA), and the Standardized Root Mean Square Residual (SRMR). Given that the χ^2^ value is influenced by the sample size, a model can be accepted when the CFI is higher than 0.90 and close to 0.95, the RMSEA is 0.08 or less, and the SRMR is 0.05 or less.

## Limitations

Our study presents some limitation. First, a retrospective study has disadvantages such as memory bias and difficulty in analysing the temporal relationship among variables. However, especially for COVID positive/hospitalized individuals and COVID positive/home isolated individuals, the retrospective study was the only way to evaluate the state of mind of the participants without the risk of overwhelming them in already difficult circumstances (i.e. being COVID-19 positive). Moreover, participants in phase 1 are especially women and this affects the generalization of our results. Secondly, the estimation of the effects size applied to determine the sample size might have led to an underestimation of the required dimension. Since no previous research has analysed this topic, further research is needed to consider a larger sample, also in other countries, in order to achieve greater generalizability of the results. Thirdly, the measures are all self-reported, thus the answers might be biased or influenced by social desirability and the measures adopted have little demonstration of validity properties. Although the measure of affective empathy has been applied in several other studies^22,23,50,57^, there is little demonstration of its validity properties and further investigation should apply validated procedures to replicate our findings. Still, the measure of acceptance of public health rules is not validated, even though it has been applied elsewhere^52^. Although this can be considered a further limitation for the generalization of the results, to the best of our knowledge there were no validated measures in literature for this kind of construct at the time of data collection. Finally, our measures of empathy and acceptance of public health rules were limited respectively to affective empathy for others at risk for COVID-19 and acceptance of the lockdown measures for COVID-19.

## Data Availability

Data are available on request from the first author.
